# Effective Management of High-Grade Left Common Carotid and Brachiocephalic Arterial Stenosis With Endovascular Stenting

**DOI:** 10.7759/cureus.13474

**Published:** 2021-02-21

**Authors:** Mohammed Salih, Osama Abdel-Hafez, Ramzi Ibrahim, Hadeel Al-ani, Feras Aloka

**Affiliations:** 1 Internal Medicine, St. Joseph Mercy Oakland Hospital, Pontiac, USA; 2 Cardiology, St. Joseph Mercy Oakland Hospital, Pontiac, USA; 3 Internal Medicine, University of Baghdad School of Medicine, Baghdad, IRQ; 4 Interventional Cardiology, St. Joseph Mercy Oakland Hospital, Pontiac, USA

**Keywords:** cardiology, interventional cardiologist, endovascular, brachiocephalic, stenosis

## Abstract

Multi-vessel disease including the brachiocephalic artery remains a relatively rare finding in atherosclerotic disease when compared to stenosis of other major vasculature. Its management presents many difficulties. Endovascular intervention is a highly preferred choice of therapy in these patients although it is dependent on operator experience. We present a case of left common carotid and brachiocephalic arterial stenosis in a patient who presented with neurological alterations that was treated with endovascular stenting. Technical difficulty was encountered during intervention but was successful.

## Introduction

Atherosclerotic disease is a systemic pathological state, which most commonly presents as carotid stenosis [1]. The involvement of the brachiocephalic artery is less common [2-4]. Brachiocephalic artery supplies a significant amount of blood to the brain as it gives rise to the right common carotid and right subclavian arteries, contributing a significant amount of the overall hemodynamic stability as an extracranial arterial system. Stenosis in the brachiocephalic artery happens less than two percent of the time in all extracranial causes of cerebrovascular insufficiency [2,5]. The outcome of this includes compromised blood flow to the right upper extremity and cerebrovascular supply [6,7]. Symptoms typically include visual alterations, pain in the right upper extremity, transient ischemic attacks (TIAs), syncopal episodes, or potentially even cerebrovascular accidents (CVA). Additionally, due to the location of the stenotic occlusive disease in the brachiocephalic artery, subclavian steal syndrome may occur [8]. We present a case of symptomatic stenosis of the left common carotid and brachiocephalic arteries that were successfully treated with endovascular stenting.

## Case presentation

An 86-year-old woman with a past medical history of a TIA presented to the emergency department complaining of nausea, left facial droop, and confusion lasting for about four hours. Physical examination was remarkable for a temperature of 98.6 F, pulse rate of 102 and regular rhythm, blood pressure of 157/84 mmHg on the left arm and 70/59 mmHg on the right arm, and a respiratory rate of 20. The neurological exam showed mild left facial droop with no other abnormalities. Examination of the other systems was unremarkable. Computed tomography (CT) scan of the head showed no acute processes. CT angiogram (CTA) of the chest was remarkable for significant high-grade stenosis in the origin of the brachiocephalic artery as well as significant stenosis of the left common carotid artery. The patient was taken to the catheterization lab where the findings seen on CTA were confirmed.

A therapeutic catheterization was to be proceeded with caution due to the challenging and problematic location of the stenotic occlusions. Using a left femoral-right brachial access, the left common carotid stenosis was encountered with difficulty in ostial engagement. Wiring this artery consisted of multiple failed attempts. Even after successful wiring, the wire position was lost multiple times in the setting of a type 3 aortic arch. Another attempt using a 3DRC guiding catheter (Adroit USA Inc., CA, USA) and a Grand Slam wire (Asahi Intecc USA, Inc., CA, USA) was successful, which was followed by balloon dilatation and 6.0 x 18 Herculink stent (Abbott, IL, USA) placement. The difficult ostial engagement was also seen with the management of the brachiocephalic artery stenosis. This consisted of multiple wire attempts until finally crossing with a Whisper wire (Abbott). After balloon dilatation, an Omnilink Abbott stent 8.0 x 29 (Abbott) was eventually placed. 

**Figure 1:**
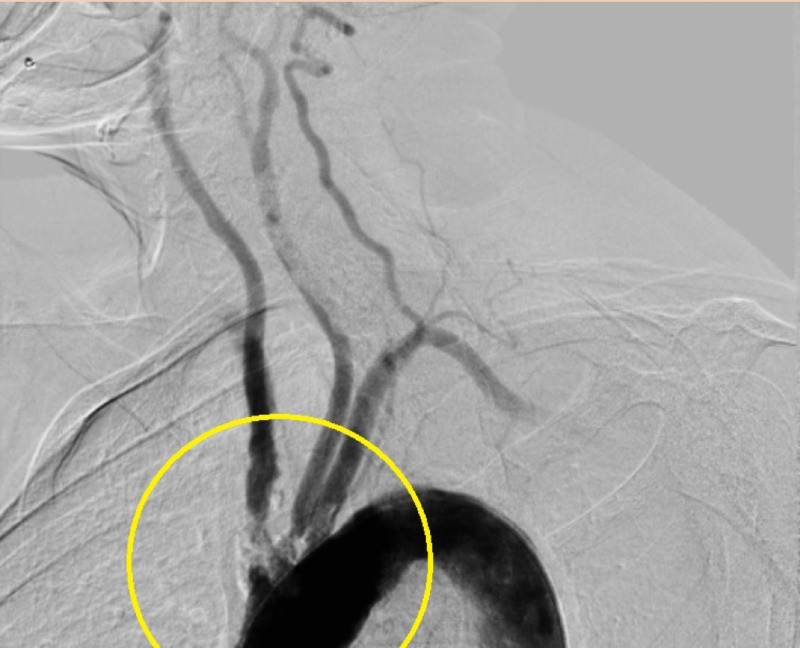
Pre-intervention arteriogram demonstrating high-grade stenosis of the left common carotid and brachiocephalic arteries.

**Figure 2:**
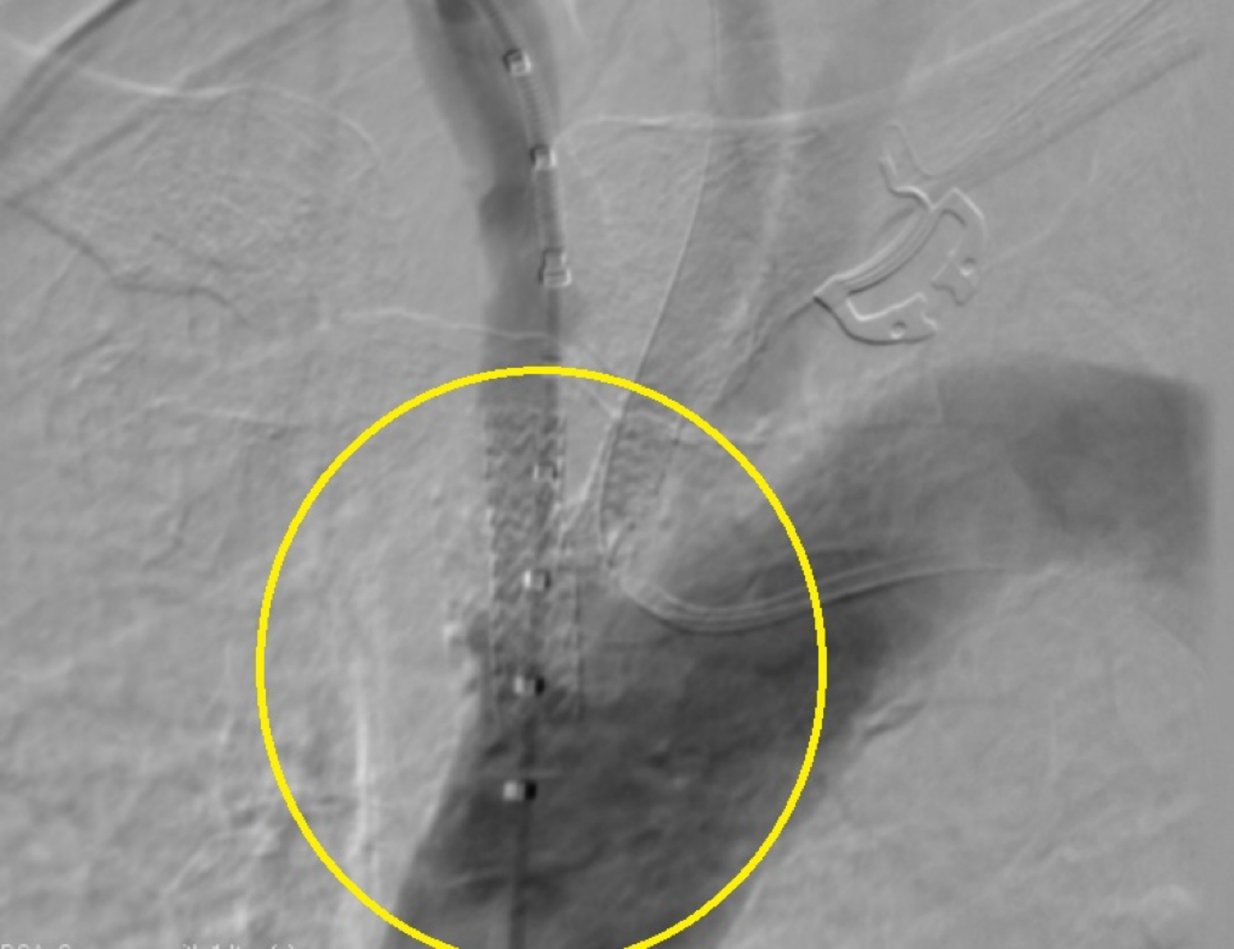
Post-intervention arteriogram demonstrating patency of the left common carotid and brachiocephalic arteries.

After the procedure, an ascending aortogram confirmed successful results with less than 10% residual stenosis in both arteries. The procedure was therefore tolerated without any post-procedural complications. Arteriograms are available pre and post-procedure to outline the findings (Figures 1, 2). Blood pressure was then taken bilaterally, resulting in equal blood pressure measurements at ~130/80 mmHg. The patient was transferred to the CCU for close monitoring and subsequently discharged on guideline-directed medical therapy.

## Discussion

The atherosclerotic disease typically involves multiple vessels simultaneously. However, the multi-vessel disease that includes brachiocephalic stenosis is a rare finding in clinical practice and an uncommon entity of atherosclerotic disease. Other potential causes of brachiocephalic stenosis include autoimmune arteritis and radiation arteritis. The brachiocephalic artery is the largest branch of the aortic arch, branching off to form the right subclavian and right common carotid arteries. This contributes to a significant amount of perfusion into the cerebral hemisphere and the right upper extremity.

Stenosis in the brachiocephalic artery is often asymptomatic; however, the altered hemodynamics can cause a spectrum of symptoms such as visual disturbances, neurological deficits, extremity pain, and more. There was an evident blood pressure discrepancy between the right and left upper extremities in our patient due to the occluded blood flow into the right arm, which can ultimately lead to ischemic gangrene if severe enough. These patients are also at a much greater risk of developing a TIA [9-10]. The right upper extremity is more commonly affected when compared to the left upper extremity due to alterations in the hemodynamics of the right subclavian artery. This extremity pain typically occurs during exertion as portrayed in a case series that confirmed its exertion dependent nature [3]. Additionally, this explains the increased risk of subclavian steal syndrome in patients with brachiocephalic artery stenosis, as a result of changing the hemodynamics of the ipsilateral vertebral artery and common carotid artery [3-5,8]. Subclavian steal phenomena may occur in one artery in about 20% of cases of brachiocephalic stenotic occlusion [4,11]. Less than 2% of these cases involve at least 2 arteries [12-13].

In our patient, hemodynamic abnormalities were detected using a CTA and confirmed during catheterization. There was evident stenosis in both the brachiocephalic and the left common carotid arteries. Treatment for severely stenotic brachiocephalic and left common carotid arteries depends significantly on operator experience and institutional resources. Options include balloon angioplasty, bypass graft revascularization, and endovascular stent placement. A catheter-based approach for proximal brachiocephalic artery stenosis is appropriate in the setting of a short stenosis/occlusive segment, as seen in our case. An important limiting factor in this procedure is operator experience. As exemplified in our case, the location of the stenosis in the left common carotid and brachiocephalic arteries, especially in the setting of type 3 aortic arch, may lead to many difficulties with ostial engagement and adequate wire positioning. The deeply set location of the brachiocephalic artery exposes many complications and risks associated with the intervention; however, exposing the operative field during an open-surgical approach has many of its own risks. An endovascular approach entails less trauma to the patient, a high success rate as portrayed in our patient, and a relatively fast recovery with convincing long-term patency rates. In cases where a catheter-based approach is unlikely to be successful as many risks are associated with this procedure such as jeopardizing the integrity of the vertebral artery, open surgical intervention may deem more appropriate. In support of this methodology for the treatment of brachiocephalic artery stenosis, studies showed varying rates of patency of the stents such as 83% at two years, up to 95% at five years' post-intervention, and up to 84% at 10 years post-intervention [14-15]. Additionally, in a case series by Hüttl et al., the technical rate of success was at 96.6% for brachiocephalic artery stenosis [16]. After the utilization of improved techniques and devices seen in interventional vascular surgery, this has become the desired method of choice compared to an open surgical approach to manage brachiocephalic arterial stenosis [17-18].

## Conclusions

Brachiocephalic artery stenosis is a rare complication seen most commonly in atherosclerotic vascular disease. Presenting symptoms vary significantly and may be a concurrent finding along with left common carotid artery stenosis. Endovascular stent placement entails significant benefits and less risk of traumatic exposure to the patient and should be considered if operator experience is present and resources are available at the institution. However, technical difficulty is a significant limitation during this procedure.
